# Genetic Variations in Nucleotide Excision Repair Pathway Genes and Risk of Allergic Rhinitis

**DOI:** 10.1155/2022/7815283

**Published:** 2022-06-03

**Authors:** Wenlong Liu, Qingxiang Zeng, Yinhui Zeng, Yiquan Tang, Renzhong Luo

**Affiliations:** Department of Otolaryngology, Guangzhou Women and Children's Medical Center, Guangzhou Medical University, Guangzhou, China

## Abstract

**Background:**

Allergic rhinitis (AR) is the most frequent inflammatory disorder in the nasal mucosa that remains unclear etiology. Mounting studies suggested that genetic instability could trigger and worsen the inflammatory response. The nucleotide excision repair (NER) system is an important pathway in maintaining the stability of the genome. Therefore, the genetic variations in NER pathway genes may have potential effects on AR risk.

**Methods:**

We evaluated the correlation between 19 candidate single nucleotide polymorphisms (SNPs) in NER pathway genes and AR susceptibility by a case-control study in a Chinese population, which contains 508 AR cases and 526 controls.

**Results:**

Three independent SNPs were identified as significantly associated with AR susceptibility, including *ERCC1* rs2298881 C > A (recessive model: adjusted odds ratios (OR) = 0.30, 95%confidence interval (CI) = 0.18–0.50, *P* < 0.0001), *ERCC1* rs11615 G > A (dominant model: adjusted OR = 1.44, 95%CI = 1.04–2.01, *P* = 0.030), and *XPC* rs2228001 A > C (dominant model: adjusted OR = 0.68, 95%CI = 0.49–0.95, *P* = 0.024). Stratified analysis showed that *ERCC1* rs2298881 AA genotype was correlated with a lower risk of AR among all the subgroups compared with rs2298881 CC/CA genotype. *XPC* rs2228001 AC/CC genotype reduced AR risk among the following subgroups: age > 60 months, clinical stage I and III.

**Conclusion:**

Our finding showed that genetic variations in NER pathway genes: *ERCC1* and *XPC* may affect the risk of AR, which will provide new insights into the genetics of AR from the perspective of DNA damage repair.

## 1. Introduction

Allergic rhinitis (AR) is defined as an immunoglobulin E- (IgE-) mediated nasal inflammatory disease and is characterized by allergic symptoms [1]. It affects more than 40% of children worldwide, and the incidence is still an increasing trend [2]. Currently, AR cannot be completely cured. AR has become a global health problem, which generates huge economic and social burdens [3, 4]. Therefore, it seems particularly important to identify the genetic risk factors and screen high-risk subjects, which could provide early intervention to prevent the occurrence of AR.

The occurrence and development of AR were attributed to the aberration of regulatory T (Treg) cells, the imbalance between Th1 and Th2 immune response, the excessive secretion of proinflammatory cytokines, and the selective accumulation in the nasal mucosa of various immune cells [5, 6]. Current studies generally believe that the interaction of environmental and genetic factors jointly determines the genesis and progression of AR [7–9]. Environmental exposure to mold stains, fungal allergens, pollens, and dust mites could initiate and exacerbate the AR [10, 11], while genetic factors may exert more significant effects on the development, severity, and treatment of AR [12, 13]. The twins' studies revealed that the inheritability of AR reaches 0.33–0.75 [14, 15].

Numerous genetic studies suggested that single nucleotide polymorphisms (SNPs) in the key genes involved in the pathogenesis of AR contribute greatly to AR susceptibility, which can be classified as interleukin, chemokines, and their corresponding receptors [16, 17]. The SNPs in the interleukin such as *IL4* [18], *IL6* [8], *IL13* [19], *IL18* [20], and *IL33* [9] have been reported associated with the risk of AR, which are the key regulators in the progression of AR.

Numerous studies suggested that there is a complex relationship between genomic instability and inflammation. The DNA damage events can activate the proinflammatory signals and then exacerbate the inflammatory response. The inflammation also contributes to DNA damage by producing nitrogen species (RNS) and reactive oxygen species (ROS). This positive feedback loop was finely regulated through a complex network of transcription factors, cellular signals, and DNA damage repair pathways [21]. DNA damage also can activate certain inflammation regulators, such as *NFκB*, a crucial transcription factor that contributes to inflammatory response greatly through facilitating transcription of proinflammatory genes [22]. Additionally, DNA damage can trigger necrosis and senescence, which also enhance the inflammatory signals by releasing many inflammatory cytokines [23, 24]. The nucleotide excision repair (NER) system is a crucial DNA repair pathway that is responsible for maintaining the integrity of the genome, and it primarily repairs bulky DNA lesions. Several core genes accomplish the repair process coordinately, including *ERCC1*, *XPA*, *XPC*, *XPD*, *XPF*, and *XPG*. Mutations and abnormal expression of these core genes may affect the NER activity and the DNA repair efficiency thus increasing genetic instability and eventually leading to cancers or inflammatory disorders. Previous studies have shown that SNPs in NER pathway genes are significantly associated with the risks of various cancers [25]. For instance, Zhuo et al. have found XPC genetic variant was susceptible to hepatoblastoma risk [26]. The association between ERCC1 SNPs and altered gastric cancer risk has been demonstrated by He et al. [27].

However, no study reports the associations between genetic variations in NER pathway genes and AR risk. Hence, we performed this current case-control study to assess this association and identified AR risk-associated genetic markers from the NER pathway, which may help to screen the individuals with a high risk of AR and make early interventions to prevent the occurrence and development of AR.

## 2. Methods

### 2.1. Study Population

In this present case-control study, 508 AR cases and 526 healthy controls were included. All the subjects are of Chinese origin and recruited from Guangzhou Women and Children's Medical Center (Guangzhou, Guangdong province, China). The diagnostic criteria were followed the Allergic Rhinitis and Impact on Asthma (ARIA) guideline criteria [28].

In brief, AR cases were recruited and diagnosed by ENT doctors according to classic nasal symptoms and positive allergens test confirmed by skin prick test or specific IgE measurement. Patients with other comorbid diseases (such as asthma and allergic dermatitis) and other systematic diseases were excluded. The severity of AR was classified according to the degree of influence level of sleep, daily activities, and work and/or school performance (mild, no influence; moderate, impair above activities; severe, severely impair above activities). The informed consent forms were signed by the guardian of all participants before the research. The study protocol was authorized by the hospital institutional review board.

### 2.2. SNP Selection and Genotyping

Potential functional SNPs among the NER core genes were selected via the dbSNP database and SNP info as described by previous research [29]. A total of 19 candidate functional SNPs in six genes of the NER pathway were identified eventually for analysis. For genotyping, the genomic DNA from the peripheral blood of all subjects was extracted and purified by applying the TIANamp Blood DNA Kit (TianGen Biotech, Beijing, China). The TaqMan real-time PCR was performed in the 384-well format for genotyping of 19 candidate SNPs among all DNA samples. The conditions of reactions were set as follow: preread stage at 60°C for 30 seconds, holding stage at 95°C 10 minutes, repeated 45 cycles each of denaturation at 95°C for 15 seconds, and annealing and extension at 60°C for 1 minute. Then, we selected standard run mode and added the reaction volume (5 *μ*L for each well in 384-well reaction plate) into the instrument. Finally, we loaded the reaction plate, then start the run. To ensure the reliability and authenticity of the results, a second-time genotyping was conducted in randomly selected 10% DNA samples. Two sets of results were 100% consistent.

### 2.3. Expression Quantitative Trait Loci (eQTL) Analysis

The eQTL is a kind of specific genetic maker that spread over genomes, which may affect gene expressions. The public databases from GTEx (Genotype-Tissue Expression) platform are usually used to analyze the correlation between genetic variants and gene expressions. Here, we performed the eQTL analysis to evaluate the bioeffect of associated SNPs on gene expression by applying the released data from GTEx Portal. The details of GTEx and analysis were reported in the previous publications [30].

### 2.4. Statistical Analysis

The goodness-of-fit *χ*^2^ test was used to evaluate whether the 19 candidate SNPs were in Hardy-Weinberg equilibrium (HWE) among control subjects. Differences in allele frequencies of selected SNPs between cases and controls were assessed using Pearson's chi-square test. The odds ratios (ORs) and 95% confidence intervals (CIs) were calculated to assess the association between 19 candidate SNPs and AR susceptibility. And the unconditional multivariate logistic regression analysis that adjusted for age and sex was conducted to calculate adjusted ORs and corresponding 95% CIs. Additionally, stratification analysis was performed in terms of age, gender, and clinical stage of subjects. The statistical analyses were performed in the SAS statistical software (version 9.4, SAS Institute, NC, USA). The result would be regarded as statistically significant when *P* value < 0.05.

## 3. Results

### 3.1. Correlations between SNPs of NER Pathway Genes and AR Susceptibility

In this present case-control study, 508 AR cases and 526 healthy controls were included for analyzing the correlations between 19 candidate SNPs and AR susceptibility. As displayed in Table 1, the observed genotype frequencies of all selected SNPs are consistent with Hardy-Weinberg equilibrium (HWE) among the controls (*P* ≥ 0.05). We discovered that *ERCC1* rs2298881 C > A was associated with decreased AR susceptibility, and carriers with rs2298881 AA genotype had significantly reduced AR risk when compared to subjects with CC/CA genotype (AOR = 0.30, 95%CI = 0.18–0.50, *P* < 0.0001, recessive model). However, another SNP in *ERCC1*: rs11615 G > A was found to be related to increased risk of AR, and subjects with GA/AA genotype have higher AR risk compared to those with GG genotype (AOR = 1.44, 95%CI = 1.04–2.01, *P* = 0.030, dominant model). In addition, we also identified *XPC* rs2228001 A > C was correlated with reduced AR susceptibility, and individuals with AC/CC genotype have a lower risk of AR compared to those with AA genotype (AOR = 0.68, 95%CI = 0.49–0.95, *P* = 0.024, dominant model). For the rest of the SNPs, no significant effect was found in AR risk (*P* ≥ 0.05).

### 3.2. Stratified Analysis

To eliminate potential effect of age, gender, and clinical stages on AR risk, we further performed the stratified analysis through adjusting these confounding factors. As displayed in Table 2, the protective effect of rs2298881 AA genotype in reducing AR risk was observed among all subgroup: age ≤ 60 months (AOR = 0.40, 95%CI = 0.20–0.77, *P* = 0.007), age > 60 (AOR = 0.46, 95%CI = 0.26–0.80, *P* = 0.007), females (AOR = 0.25, 95%CI = 0.12–0.55, *P* = 0.0004), males (AOR = 0.35, 95%CI = 0.18–0.68, *P* = 0.002), clinical I (AOR = 0.44, 95%CI = 0.23–0.85, *P* = 0.015), clinical II (AOR = 0.24, 95%CI = 0.12–0.46, *P* < 0.0001), and clinical III (AOR = 0.39, 95%CI = 0.20–0.79, *P* = 0.009). However, the significant risk effect of rs11615 G > A in AR was not found among all subgroups (all *P* > 0.05). And *XPC* rs2228001 AC/CC genotype decreased the AR risk among following subgroups: age > 60 (AOR = 0.58, 95%CI = 0.37–0.93, *P* = 0.022), clinical I (AOR = 0.44, 95%CI = 0.28–0.69, *P* = 0.0004), and clinical III (AOR = 0.51, 95%CI = 0.31–0.82, *P* = 0.006).

### 3.3. eQTL Analysis

**Figure 1:**
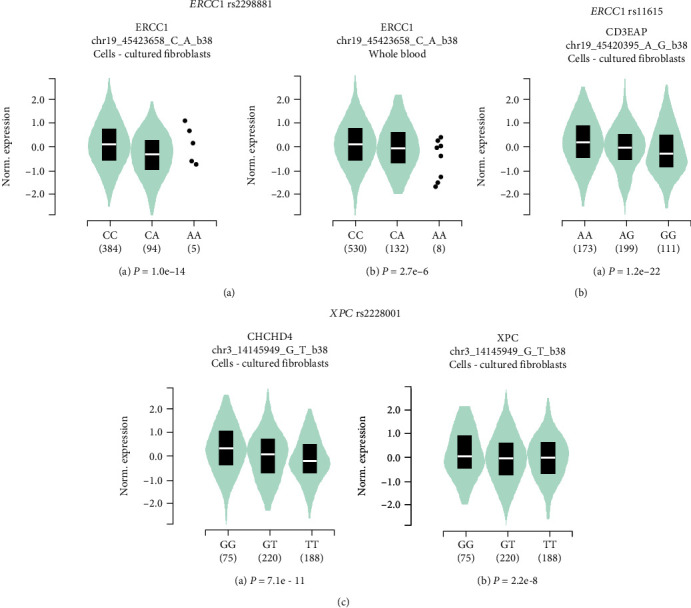
Expression quantitative trait loci (eQTL) analysis of the allergic rhinitis risk factors *ERCC1* rs2298881 C > A, *ERCC1* rs11615 G > A, and *XPC* rs2228001 A > C. (A) *ERCC1* rs2298881 C > A genotype-based mRNA expression alteration of *ERCC1* gene in the cells-cultured fibroblasts (a) and whole blood (b); (B) *ERCC1* rs11615 G > A genotype-based mRNA expression change of *CD3EAP* gene in the cell-cultured fibroblasts; (C) *XPC* rs2228001 A > C genotype-based mRNA expression change of *CHCHD4* (a) and *XPC* (b) genes in the cells-cultured fibroblasts.

To further explore the potential biologic effects of the significant SNPs on the adjacent gene expressions and the possible mechanism by which these significant SNPs modify AR susceptibility, we performed the eQTL analysis from the GTEx platform. We discovered that the rs2298881 A allele was significantly related to decreased mRNA expression of *ERCC1* in the cell-cultured fibroblasts and whole blood (Figure 1(a)). However, another significant SNP in *ERCC1*: rs11615 G > A was not associated with the mRNA level of *ERCC1* but affected the gene expression of *CD3EAP*. The *CD3EAP* mRNA with *ERCC1* rs11615 G allele was significantly lower than those with *ERCC1* rs11615 A allele in the cell-cultured fibroblasts (Figure 1(b)). For the SNP rs2228001, the T allele was found to be significantly associated with lower mRNA levels of *CHCHD4* and *XPC* compared to those with the G allele in the cell-cultured fibroblasts (Figure 1(c)).

## 4. Discussion

To explore the effects of SNP in NER pathway genes on AR susceptibility, we performed this current case-control research in the Chinese population which comprehensively assessed the association between 19 functional SNPs in 6 NER core genes and AR risk. Our results showed that two SNPs (rs2298881 C > A and rs11615 G > A) in the *ERCC1* gene and one SNP (rs2228001 A > C) in the *XPC* gene were significantly associated with AR risk. To the best of our knowledge, this research is the first study that systematically evaluated the relationship between multiple functional SNPs in NER pathway genes and AR susceptibility. Our findings may help screen the high-risk groups and make early interventions of AR, which will greatly reduce its morbidity.

Although being subject of extensive study, the pathogenesis of AR is still poorly understood, which might attribute to its intricate etiology that involves the complex interactions of genetic and environmental factors [31, 32]. However, mounting studies have shown that genetic factors exert significant effects on the genesis, severity, and response to treatment of AR [33]. Numerous studies revealed that SNP in certain pivotal genes involved in the pathology of AR, such as interleukin, chemokine, and their receptor coding genes, will modify the susceptibility of AR. For example, *IL4* and *IL13* are known for their key role in the pathogenesis of AR. One SNP rs2243250 C > T located in the promoter region of *IL4* was shown associated with an increased risk of AR. The further study uncovered that this SNP can upregulate the expression of *IL4* and increase the plasma IgE subsequently, which will exacerbate the symptoms of AR eventually [33]. Furthermore, another SNP rs20541 A > C located in exon 4 of *IL13* was demonstrated increasing the risk of AR in Asian populations significantly [19]. Functional studies discovered that SNP rs20541 A > C results in an amino acid change from glutamine to arginine, which is involved in the transcriptional activity and increases the activity or signaling of *IL13*. It was reported that rs20541 A allele was related to higher serum *IL13* and IgE levels, which contributed to an increase of eosinophil counts and increased risk of AR [34, 35]

Despite it being widely believed that DNA damage was one of the most common events in cancer, however, growing studies showed that genomic instability also triggers inflammatory responses. Previous researches have shown that some crucial transcription factors were activated during inflammation, such as *HMGB1* and *NFκB*, which caused and aggravate inflammatory responses by augmenting the expression of downstream proinflammatory cytokines [36, 37]. In addition, DNA damage-driven senescence or apoptosis also exacerbates the inflammatory response by releasing various inflammatory factors or other ways [38, 39]. Therefore, genetic instability may be an important source of proinflammatory signals and promote the development of inflammation.

The NER pathway is the primary repair mechanism for DNA damage, which plays an important role in maintaining genomic stability and preventing the occurrence of diseases, such as various cancers and inflammations. The NER process is completed collaboratively by the NER machinery that is composed of several crucial enzymes: *ERCC-1* and *XPF* encode 5′ endonucleases, *XPA*, and *XPC* function as damage recognition, *XPD* gene encodes the helicase and the *XPG* for 3′ endonuclease. The abnormity of these NER core genes may affect the DNA repair efficiency and increase the probability of genome instability, therefore, increasing the risk of inflammation. One study conducted by Gungor et al. suggested that the reduced NER activity contributed to the LPS-induced acute pulmonary inflammation [40]. Horio et al. showed that *XPA*-deficient mice developed stronger and longer-lasting acute inflammation than wild-type mice after irradiation with UVB [41]. A more recent study also found that PM2.5 promoted a stronger inflammatory response in *XPC* knock-out mice compared with wild-type mice [42]. However, polymorphisms in NER core genes may result in the variation of expression and activity of these genes and DNA repair efficiency, which may modify the susceptibility of various disorders. Numerous studies have shown significant associations between SNP of NER core genes and multiple cancers susceptibilities [25, 43, 44]. Whereas, scarcely any study reported this association in inflammatory disease, including AR.

In this current study, we as a vanguard to first comprehensively assessed the association between 19 SNPs in 6 NER core genes and AR susceptibility. Here, we identified three SNPs: *ERCC1* rs2298881 C > A, *ERCC1* rs11615 G > A, and *XPC* rs2228001 A > C were significantly associated with AR susceptibility. Detailed *ERCC1* rs2298881 AA genotype decreased AR risk significantly when compared with CC/CA genotype. However, *ERCC1* rs11615 GA/AA genotype was found to increase AR risk compared with the GG genotype. And *XPC* rs2228001 AC/CC genotype also reduced the risk of AR compared with those with AA genotype. The stratification analysis further showed that the protective effect of *ERCC1* rs2298881 AA genotype was observed among all subgroups: age ≤ 60 months, age > 60, females, males, clinical I, clinical II, and clinical III. But the risk effect of *ERCC1* rs11615 GA/AA genotype was disappeared among all subgroups. Maybe these confounding factors (age, gender, and clinical stages) have certain potential effects on AR risk, or it is just a chance finding attributed to the relatively small sample size in the stratified analysis. And carriers with *XPC* rs2228001 AC/CC genotype had a lower risk of AR compared with those with AA genotype in the following subgroups: age > 60, clinical I, and clinical III. The proteins of *ERCC1* and *XPC* play an essential role during the NER process because of their excision and damage recognition ability. The *ERCC1* protein can form a heterodimer with the *XPF*, and the complex that catalyzes the incision of 5′ phosphodiester backbone around the DNA damage sites was an indispensable component in the NER. And the protein of *XPC* can also form the *XPC–HR23B* complex by binding to the *HR23B* tightly, which plays a key role in the early DNA damage recognition and the recruitment of the transcription factor IIH to the sites of DNA lesions during the NER. Polymorphisms in the *ERCC1* and *XPC* genes may influence the genomic stability then modify the disease risk, especially cancers. For example, He et al. showed that the rs2298881 C allele and rs11615 A allele increased the susceptibility of gastric cancer [45]. And Malik et al. suggested that rs2228001 A > C may change the C-terminus functional preferences and structure of *XPC*, then contribute to the breast cancer risk [46]. However, no study reports the association between the SNP in NER pathway genes and AR susceptibility. Here, we first reported that SNP in *ERCC1* (rs2298881 C > A and rs11615 G > A) and *XPC* (rs2228001 A > C) genes modify the risk of AR.

How these associated SNP modify the AR susceptibility? Interestingly, a recent study has introduced compelling evidence about shorter telomere lengths (TLs) among patients with AR. TLs as biomarkers of aging are prompted shortening by raised inflammation [47]. As a DNA repair protein, the ERCC1 may have influenced TLs wane procedure, such as sheltering them from homologous recombination [48], mediating a suppressive role in TLs maintenance by controlling the critical factor, TRF2 [49]. Because of the significant difference of ERCC1 genetic variation between AR and controls in our finding, which, therefore denotes the difference in TLs between them.

To further explore the functional effects of these significant SNPs on the expression of adjacent genes, then dope out the possible mechanisms by which the associated SNPs affect the AR risk, the eQTL analysis was carried out. Our results showed that rs2298881 A allele was significantly associated with lower mRNA expression of *ERCC1* in the cell-cultured fibroblasts and whole blood, and rs11615 G allele was found to reduce the mRNA level of *CD3EAP*. Regarding rs2228001, the T allele was related to decreased expression of *CHCHD4* and *XPC*. Maybe these SNP-base expression changes of neighboring genes contribute to the modification of genotype-base AR risk. However, further study is still needed to illuminate the exact underlining mechanisms. Although in the initial stage, our study provides new insights into the modification of AR risk by the NER pathway gene variants.

There are several concomitant limitations in this study. First, the present case-control study is hospital-based, so the selection bias is ineluctable. Second, the sample size enrolled in this study remained moderate, however, it was relatively small for stratified analysis, which may whittle the statistical power and reduce the reliability of the conclusions. Third, although we have made a comprehensive assessment on 19 SNP of NER pathway genes, other potentially functional SNP should be assessed. Fourth, environmental factors should be considered, as the etiology of AR involves complex interactions between multiple genetic and environmental factors. Fifth, the conclusions from this research may not apply to any ethnic group other than the Chinese, because of the Chinese origin of all participants. Sixth, mechanism studies should be included, which will further elucidate the underlying mechanism by which genetic variations in NER pathway genes modify the AR risk. Seventh, most of AR cases in this study were perennial and caused by indoor allergen, especially house dust mite (>90%). Therefore, despite that there may be significant differences in pathogenesis between perennial and seasonal rhinitis, we believe that there may be less effect on our study. However, further study on seasonal rhinitis was also needed.

## 5. Conclusions

In summary, this current research was the first case-control study to systematically evaluate the effects of SNPs in NER-associated genes on AR risk. Our findings showed that in Chinese children, genetic variations in *ERCC1* and *XPC* genes influence AR susceptibility significantly. Well-designed studies with a large sample size involving different ethnicities should be performed to verify our conclusions in the future. Further, the potentially exact mechanisms that *ERCC1* and *XPC* genetic variants modify AR susceptibility should be revealed by further functional studies.

## Figures and Tables

**Table 1:** Association between polymorphisms in nucleotide excision repair pathway genes and allergic rhinitis susceptibility.

Gene	SNP	Allele		Case (*N* = 508)			Control (*N* = 526)			Adjusted OR ^a^	*P* ^a^	Adjusted OR^b^	*P* ^b^	HWE
		A	B	AA	AB	BB	AA	AB	BB	(95% CI)		(95% CI)		
*ERCC1*	rs2298881	C	A	225	231	52	205	231	90	0.89 (0.64-1.24)	0.493	**0.30 (0.18-0.50)**	**<0.0001**	0.075
*ERCC1*	rs3212986	C	A	199	242	67	243	222	61	1.38 (0.99-1.92)	0.060	1.14 (0.69-1.87)	0.617	0.346
*ERCC1*	rs11615	G	A	261	204	43	297	185	44	**1.44 (1.04-2.01)**	**0.030**	0.99 (0.54-1.81)	0.975	0.052
*XPA*	rs1800975	T	C	142	277	89	126	276	124	0.74 (0.51-1.07)	0.106	0.78 (0.52-1.17)	0.277	0.257
*XPA*	rs3176752	G	T	357	138	13	395	123	8	1.01 (0.70-1.46)	0.942	1.48 (0.49-4.53)	0.489	0.653
*XPC*	rs2228001	A	C	227	220	61	208	251	67	**0.68 (0.49-0.95)**	**0.024**	0.88 (0.53-1.47)	0.632	0.517
*XPC*	rs2228000	C	T	184	247	77	206	249	71	1.12 (0.80-1.56)	0.527	1.44 (0.90-2.31)	0.131	0.756
*XPC*	rs2607775	C	G	463	45	0	473	53	0	0.90 (0.50-1.60)	0.718	/	/	0.224
*XPC*	rs1870134	G	C	317	161	30	335	165	26	1.18 (0.84-1.65)	0.338	1.27 (0.62-2.59)	0.514	0.335
*XPC*	rs2229090	G	C	168	249	91	196	252	78	1.21 (0.86-1.70)	0.276	1.28 (0.82-1.98)	0.274	0.837
*XPD*	rs3810366	G	C	148	265	95	146	251	129	1.05 (0.73-1.51)	0.782	0.87 (0.58-1.30)	0.494	0.306
*XPD*	rs238406	G	T	114	266	128	152	248	126	1.22 (0.84-1.79)	0.294	0.94 (0.64-1.38)	0.753	0.209
*XPD*	rs13181	T	G	436	69	3	456	66	4	1.21 (0.75-1.95)	0.447	2.90 (0.51-16.32)	0.228	0.351
*XPF*	rs2276466	C	G	289	195	24	299	195	32	0.94 (0.67-1.31)	0.709	0.74 (0.35-1.58)	0.441	0.978
*XPG*	rs2094258	C	T	214	231	63	201	249	76	0.78 (0.56-1.08)	0.132	0.89 (0.55-1.44)	0.640	0.937
*XPG*	rs751402	C	T	205	235	68	209	235	82	0.85 (0.61-1.19)	0.347	1.04 (0.66-1.64)	0.851	0.241
*XPG*	rs2296147	T	C	321	165	22	341	166	19	1.27 (0.90-1.79)	0.172	0.79 (0.34-1.86)	0.589	0.828
*XPG*	rs1047768	T	C	270	200	38	309	189	28	1.33 (0.96-1.85)	0.092	1.29 (0.68-2.48)	0.438	0.897
*XPG*	rs873601	G	A	124	244	140	132	269	125	0.97 (0.66-1.42)	0.865	1.45 (1.00-2.12)	0.053	0.598

OR: odds ratio; CI: confidence interval; HWE: Hardy–Weinberg equilibrium. The results were in bold if the 95% CI excluded 1 or *P* < 0.05. ^a^Adjusted for age and sex for dominant model. ^b^Adjusted for age and sex for recessive model.

**Table 2:** Stratification analysis of *ERCC1* and *XPC* genotypes with allergic rhinitis risk.

Variables	*ERCC1* rs2298881 (case/control)		AOR (95% CI)	*P* ^a^	*ERCC1* rs11615 (case/control)		AOR (95% CI)	*P* ^a^	*XPC* rs2228001 (case/control)		AOR (95% CI)	*P* ^a^
	CC/CA	AA			GG	GA/AA			AA	AC/CC		
Age, month												
≤60	153/356	11/66	**0.40 (0.20-0.77)**	**0.007**	78/237	86/185	1.40 (0.97-2.01)	0.072	73/175	91/247	0.89 (0.62-1.28)	0.522
>60	303/80	41/24	**0.46 (0.26-0.80)**	**0.007**	183/60	161/44	1.20 (0.77-1.87)	0.419	154/33	190/71	**0.58 (0.37-0.93)**	**0.022**
Sex												
Females	235/205	22/36	**0.25 (0.12-0.55)**	**0.0004**	132/129	125/112	1.37 (0.84-2.22)	0.209	120/98	137/143	0.66 (0.40-1.08)	0.094
Males	221/231	30/54	**0.35 (0.18-0.68)**	**0.002**	129/168	122/117	1.52 (0.97-2.39)	0.068	107/110	144/175	0.70 (0.44-1.10)	0.120
Clinical stages												
I	130/436	17/90	**0.44 (0.23-0.85)**	**0.015**	80/297	67/229	1.24 (0.79-1.95)	0.341	79/208	68/318	**0.44 (0.28-0.69)**	**0.0004**
II	208/436	19/90	**0.24 (0.12-0.46)**	**<0.0001**	108/297	119/229	1.47 (0.99-2.18)	0.058	84/208	143/318	0.89 (0.59-1.34)	0.587
III	118/436	16/90	**0.39 (0.20-0.79)**	**0.009**	73/297	61/229	1.24 (0.77-2.00)	0.369	64/208	70/318	**0.51 (0.31-0.82)**	**0.006**

CI: confidence interval; AOR: adjusted odds ratio. The results were in bold if the 95% CI excluded 1 or *P* < 0.05. ^a^Obtained in logistic regression models with adjustment for age and sex omitting the corresponding stratification factor.

## Data Availability

The datasets used and analyzed during the current study are all available from the corresponding author.

## References

[1] Wallace D. V., Dykewicz M. S., Bernstein D. I. (2008). The diagnosis and management of rhinitis: an updated practice parameter. *The Journal of Allergy and Clinical Immunology*.

[2] Lancet T. (2008). Allergic rhinitis: common, costly, and neglected. *Lancet*.

[3] Settipane R. A., Charnock D. R. (2007). Epidemiology of rhinitis: allergic and nonallergic. *Clinical Allergy and Immunology*.

[4] Price D., Smith P., Hellings P. (2015). Current controversies and challenges in allergic rhinitis management. *Expert Review of Clinical Immunology*.

[5] Pawankar R., Mori S., Ozu C., Kimura S. (2011). Overview on the pathomechanisms of allergic rhinitis. *Asia Pacific Allergy*.

[6] Botturi K., Lacoeuille Y., Cavailles A., Vervloet D., Magnan A. (2011). Differences in allergen-induced T cell activation between allergic asthma and rhinitis: role of CD28, ICOS and CTLA-4. *Respiratory Research*.

[7] Meng Y., Wang C., Zhang L. (2019). Recent developments and highlights in allergic rhinitis. *Allergy*.

[8] Zhao N., Liu H. J., Sun Y. Y., Li Y. Z. (2016). Role of interleukin-6 polymorphisms in the development of allergic rhinitis. *Genet Mol Res*.

[9] Ran H., Xiao H., Zhou X., Guo L., Lu S. (2020). Single-nucleotide polymorphisms and haplotypes in the interleukin-33 gene are associated with a risk of allergic rhinitis in the Chinese population. *Experimental and Therapeutic Medicine*.

[10] Skoner D. P. (2001). Allergic rhinitis: definition, epidemiology, pathophysiology, detection, and diagnosis. *The Journal of Allergy and Clinical Immunology*.

[11] Dunlop J., Matsui E., Sharma H. P. (2016). Allergic rhinitis: environmental determinants. *Immunology and Allergy Clinics of North America*.

[12] Vercelli D. (2003). Genetic polymorphism in allergy and asthma. *Current Opinion in Immunology*.

[13] Andiappan A. K., Nilsson D., Halldén C. (2013). Investigating highly replicated asthma genes as candidate genes for allergic rhinitis. *BMC Medical Genetics*.

[14] Feijen M., Gerritsen J., Postma D. S. (2000). Genetics of allergic disease. *British Medical Bulletin*.

[15] Fagnani C., Annesi-Maesano I., Brescianini S. (2008). Heritability and shared genetic effects of asthma and hay fever: an Italian study of young twins. *Twin Research and Human Genetics*.

[16] Davila I., Mullol J., Ferrer M. (2009). Genetic aspects of allergic rhinitis. *Journal of Investigational Allergology & Clinical Immunology*.

[17] Fuertes E., Brauer M., MacIntyre E. (2013). Childhood allergic rhinitis, traffic-related air pollution, and variability in the _GSTP1_ , _TNF_ , _TLR2_ , and _TLR4_ genes: results from the TAG Study. *The Journal of Allergy and Clinical Immunology*.

[18] Micheal S., Minhas K., Ishaque M., Ahmed F., Ahmed A. (2013). IL-4 gene polymorphisms and their association with atopic asthma and allergic rhinitis in Pakistani patients. *Journal of Investigational Allergology & Clinical Immunology*.

[19] Chen M. L., Zhao H., Huang Q. P., Xie Z. F. (2018). Single nucleotide polymorphisms of IL-13 and CD14 genes in allergic rhinitis: a meta-analysis. *European Archives of Oto-Rhino-Laryngology*.

[20] Tharabenjasin P., Pabalan N., Jarjanazi H., Poachanukoon O. (2020). Influence of polymorphisms in the interleukin-18 gene on allergic rhinitis: a meta-analysis. *International Archives of Allergy and Immunology*.

[21] Kay J., Thadhani E., Samson L., Engelward B. (2019). Inflammation-induced DNA damage, mutations and cancer. *DNA Repair*.

[22] Tadie J. M., Bae H. B., Deshane J. S. (2012). Toll-like receptor 4 engagement inhibits adenosine 5'-monophosphate-activated protein kinase activation through a high mobility group box 1 protein-dependent mechanism. *Molecular Medicine*.

[23] Davidovich P., Kearney C. J., Martin S. J. (2014). Inflammatory outcomes of apoptosis, necrosis and necroptosis. *Biological Chemistry*.

[24] Rodier F., Coppé J. P., Patil C. K. (2009). Persistent DNA damage signalling triggers senescence-associated inflammatory cytokine secretion. *Nature Cell Biology*.

[25] Zhou C., Wang Y., He L. (2021). Association between NER pathway gene polymorphisms and neuroblastoma risk in an eastern Chinese population. *Molecular Therapy-Oncolytics*.

[26] Zhuo Z., Miao L., Hua W. (2021). Genetic variations in nucleotide excision repair pathway genes and hepatoblastoma susceptibility. *International Journal of Cancer*.

[27] He J., Zhuo Z. J., Zhang A. (2018). Genetic variants in the nucleotide excision repair pathway genes and gastric cancer susceptibility in a southern Chinese population. *Cancer Management and Research*.

[28] Shirkani A., Mansouri A., Abbaszadegan M. R., Faridhosseini R., Jabbari Azad F., Gholamin M. (2017). <i>GATA3</i> gene polymorphisms associated with allergic rhinitis in an Iranian population. *Reports of Biochemistry & Molecular Biology*.

[29] He J., Qiu L. X., Wang M. Y. (2012). Polymorphisms in the XPG gene and risk of gastric cancer in Chinese populations. *Human Genetics*.

[30] Carithers L. J., Moore H. M. (2015). The genotype-tissue expression (GTEx) project. *Biopreservation and Biobanking*.

[31] Wang J., Li B., Yu W. (2014). Rhinitis symptoms and asthma among parents of preschool children in relation to the home environment in Chongqing, China. *PLoS One*.

[32] Xu Y., Zhang J. X. (2015). ADAM33 polymorphisms and susceptibility to allergic rhinitis: a meta-analysis. *European Archives of Oto-Rhino-Laryngology*.

[33] Shirkani A., Mansouri A., Farid Hosseini R. (2019). The role of interleukin-4 and 13 gene polymorphisms in allergic rhinitis: a case control study. *Reports of Biochemistry & Molecular Biology*.

[34] Arima K., Umeshita-Suyama R., Sakata Y. (2002). Upregulation of IL-13 concentration in vivo by the _IL13_ variant associated with bronchial asthma. *The Journal of Allergy and Clinical Immunology*.

[35] Vladich F. D., Brazille S. M., Stern D., Peck M. L., Ghittoni R., Vercelli D. (2005). IL-13 R130Q, a common variant associated with allergy and asthma, enhances effector mechanisms essential for human allergic inflammation. *The Journal of Clinical Investigation*.

[36] Yang H., Tracey K. J. (2010). Targeting HMGB1 in inflammation. *Biochimica et Biophysica Acta*.

[37] Ba X., Bacsi A., Luo J. (2014). 8-Oxoguanine DNA glycosylase-1 augments proinflammatory gene expression by facilitating the recruitment of site-specific transcription factors. *Journal of Immunology*.

[38] Rodier F., Muñoz D. P., Teachenor R. (2011). DNA-SCARS: distinct nuclear structures that sustain damage-induced senescence growth arrest and inflammatory cytokine secretion. *Journal of Cell Science*.

[39] Miwa K., Asano M., Horai R., Iwakura Y., Nagata S., Suda T. (1998). Caspase 1-independent IL-1*β* release and inflammation induced by the apoptosis inducer Fas ligand. *Nature Medicine*.

[40] Güngör N., Haegens A., Knaapen A. M. (2010). Lung inflammation is associated with reduced pulmonary nucleotide excision repair in vivo. *Mutagenesis*.

[41] Horio T., Miyauchi-Hashimoto H., Kuwamoto K., Horiki S., Okamoto H., Tanaka K. (2001). Photobiologic and photoimmunologic characteristics of XPA gene-deficient mice. *The Journal of Investigative Dermatology. Symposium Proceedings*.

[42] de Oliveira Alves N., Pereira G. M., Di Domenico M. (2020). Inflammation response, oxidative stress and DNA damage caused by urban air pollution exposure increase in the lack of DNA repair XPC protein. *Environment International*.

[43] Liu J., He C., Xing C., Yuan Y. (2014). Nucleotide excision repair related gene polymorphisms and genetic susceptibility, chemotherapeutic sensitivity and prognosis of gastric cancer. *Mutation Research*.

[44] Li Y. K., Xu Q., Sun L. P. (2020). Nucleotide excision repair pathway gene polymorphisms are associated with risk and prognosis of colorectal cancer. *World Journal of Gastroenterology*.

[45] He J., Xu Y., Qiu L. X. (2012). Polymorphisms in ERCC1 and XPF genes and risk of gastric cancer in an eastern Chinese population. *PloS One*.

[46] Malik S. S., Zia A., Rashid S. (2020). XPC as breast cancer susceptibility gene: evidence from genetic profiling, statistical inferences and protein structural analysis. *Breast Cancer*.

[47] Tung K. T., Wong R. S., Tsang H. W. (2021). Impact of snoring on telomere shortening in adolescents with atopic diseases. *Genes*.

[48] Vannier J. B., Depeiges A., White C., Gallego M. E. (2009). ERCC1/XPF protects short telomeres from homologous recombination in Arabidopsis thaliana. *PLoS Genetics*.

[49] Wu Y., Mitchell T. R., Zhu X. D. (2008). Human XPF controls TRF2 and telomere length maintenance through distinctive mechanisms. *Mechanisms of Ageing and Development*.

